# Supplementary material for: PATJ deficiency leads to cystic kidney disease and related ciliopathies
Source: HGG Adv. 2025 Sep 9;7(1):100514. doi: 10.1016/j.xhgg.2025.100514 (PMC12512994; doi:10.1016/j.xhgg.2025.100514)
Supplement: Document S1. Figures S1–S4 [file mmc1.pdf]

HGGA, Volume 7

Supplemental information

PATJ deficiency leads to cystic

kidney disease and related ciliopathies

Daniel Epting, Daniela A. Braun, Eva Decker, Elisabeth Ott, Tobias Eisenberger, Nadine Bachmann, Pavel Nedvetsky, Michael P. Krahn, Friedhelm Hildebrandt, and Carsten Bergmann

Supplemental information

Table of content:

Figure S1. Analyses of Patj knockdown efficiency and knockdown with *patj*-MO2 results in ciliopathy-associated phenotypes in zebrafish

Figure S2. Analyses of ciliogenesis in the pronephric tubules of Patj knockdown embryos reveals unaffected cilia formation

Figure S3. Generation of CRISPR/Cas9-induced *patj, mpdz* and *patj;mpdz* zebrafish mutants

Figure S4. Analyses of cilia formation and ciliopathy-associated phenotypes in CRISPR/Cas9-induced *patj, mpdz* and *patj;mpdz* zebrafish mutants

Supplemental Materials and Methods

Supplemental References

Figure S1

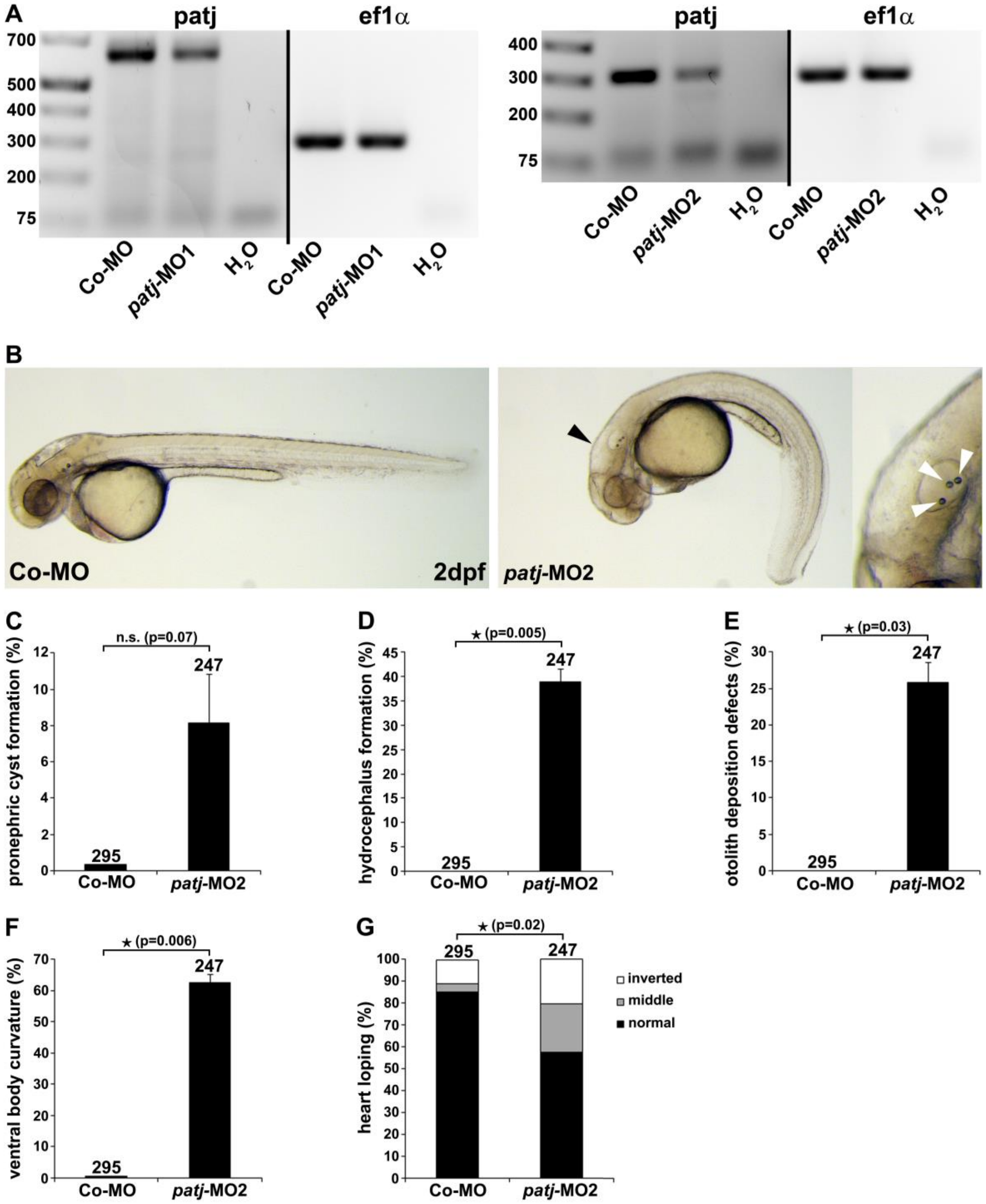

Figure S1. Analyses of Patj knockdown efficiency and knockdown with *patj*-MO2 results in ciliopathy-associated phenotypes in zebrafish

(A) RT-PCR reveals knockdown efficiency of splice-blocking MOs *patj*-MO1 and *patj*-MO2. Injection of either *patj*-MO1 or *patj*-MO2 results in significant reduced *patj* PCR-product level in the respective Patj morphant embryos compared to the control. H_2O served as negative control and *ef1α* as loading control. (B) Representative bright-field images of Co-MO and *patj*-MO2 injected embryos at 2dpf. In comparison to Co-MO injected embryos, injection of *patj*-

MO2 leads to hydrocephalus formation (black arrowhead), ventral body curvature and otolith deposition defects (white arrowheads indicate otoliths shown in a higher magnification of the otic vesicle; a typical defect is the absence of otoliths or the presence of one or three otoliths instead of two). Embryos are shown from lateral with anterior to the left. (C-G) Quantification of pronephric cyst formation (C), hydrocephalus formation (D), otolith deposition defects (E), ventral body curvature (F) and altered heart looping (analyzed as normal, middle (unlooped), inverted) (G) of embryos injected with Co-MO or *patj*-MO2 at 2dpf; number of embryos used for analyses are shown above each respective bar.

Figure S2

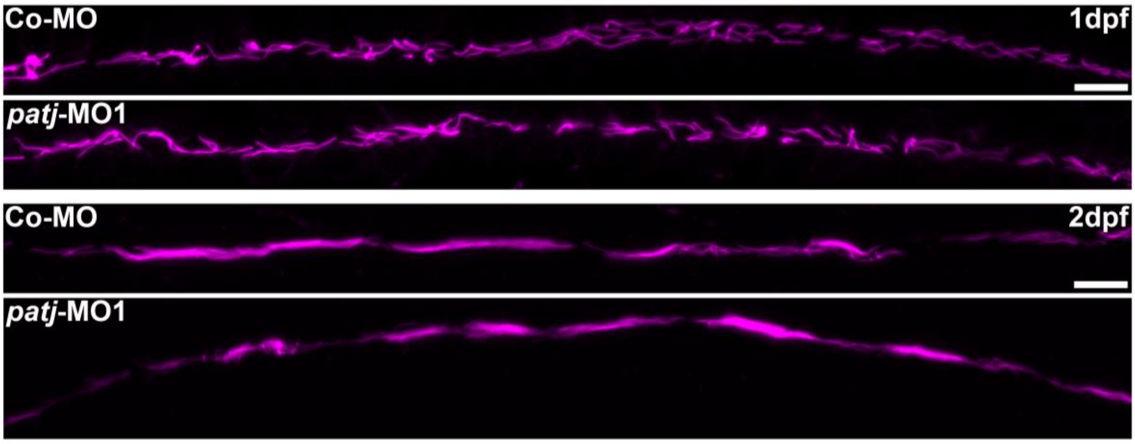

Figure S2. Analyses of ciliogenesis in the pronephric tubules of Patj knockdown embryos reveals unaffected cilia formation

Representative confocal images of the pronephric tubule of Co-MO and *patj*-MO1 injected embryos at 1 and 2dpf immunostained with anti-acetylated Tubulin as a ciliary marker. Scale bar: 10µm.

Figure S3

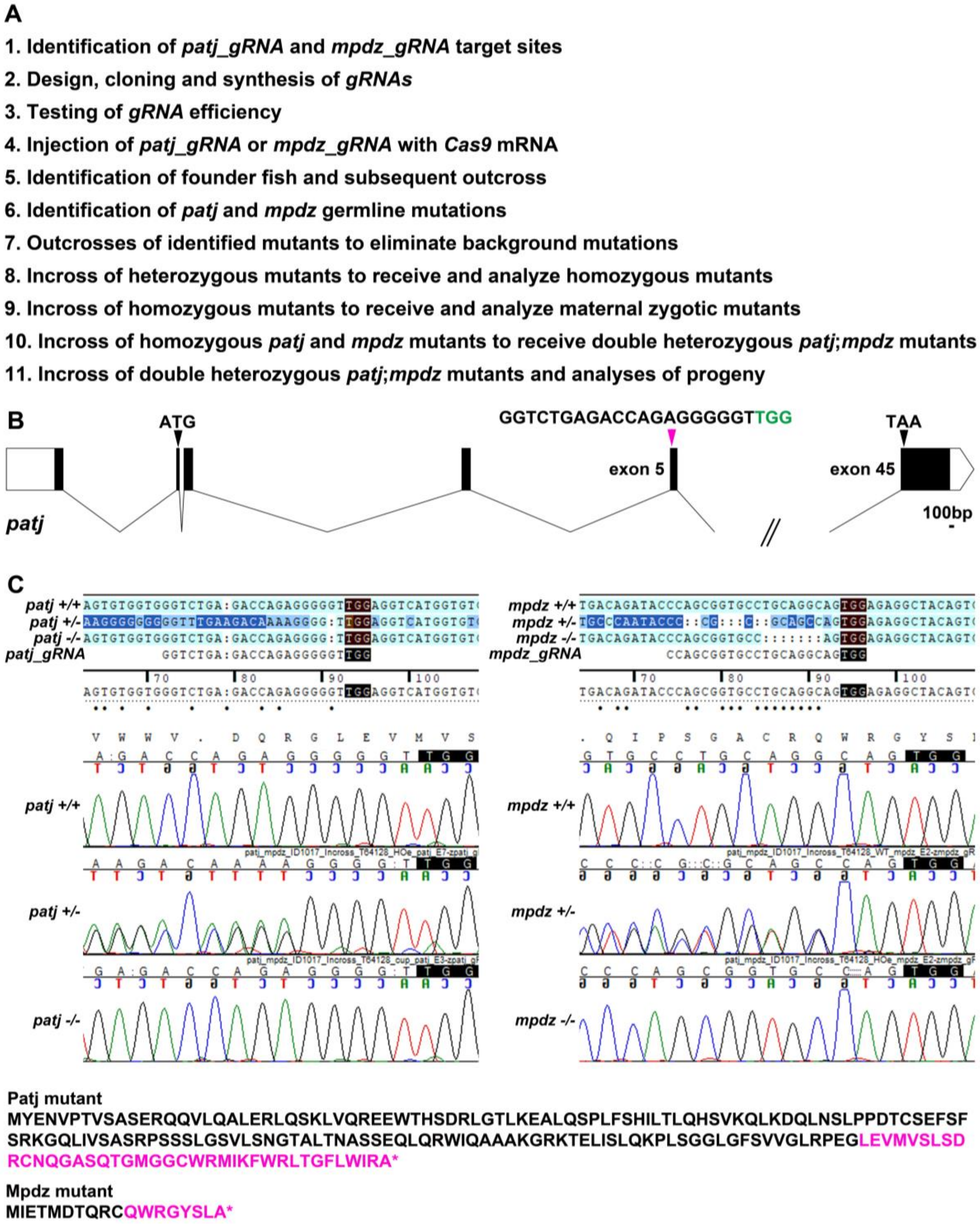

Figure S3. Generation of CRISPR/Cas9-induced *patj*, *mpdz* and *patj;mpdz* zebrafish mutants

(A) Flowchart summarizing the generation of CRISPR/Cas9-induced *patj* and *mpdz* single mutants and *patj;mpdz* double mutants in zebrafish. (B) Exon-intron structure of zebrafish *patj* (ENSDART00000132829.3) with the start codon ATG and stop codon TAA (black arrowheads) and the *patj_gRNA* target site (magenta arrowhead). (C) Sanger sequencing confirmed the deletion of nucleotide G in heterozygous and homozygous *patj* zebrafish mutants

compared to the respective control. Sanger sequencing confirmed the deletion of nucleotides TGCAGGC in heterozygous and homozygous *mpdz* zebrafish mutants compared to the respective control. The deletion of the nucleotide G in exon 5 of zebrafish *patj* results in a frameshift and premature stop codon in Patj mutants. The deletion of the nucleotides TGCAGGC in zebrafish *mpdz* (ENSDART00000160523.2) results in a frameshift and premature stop codon in Mpdz mutants.

Figure S4

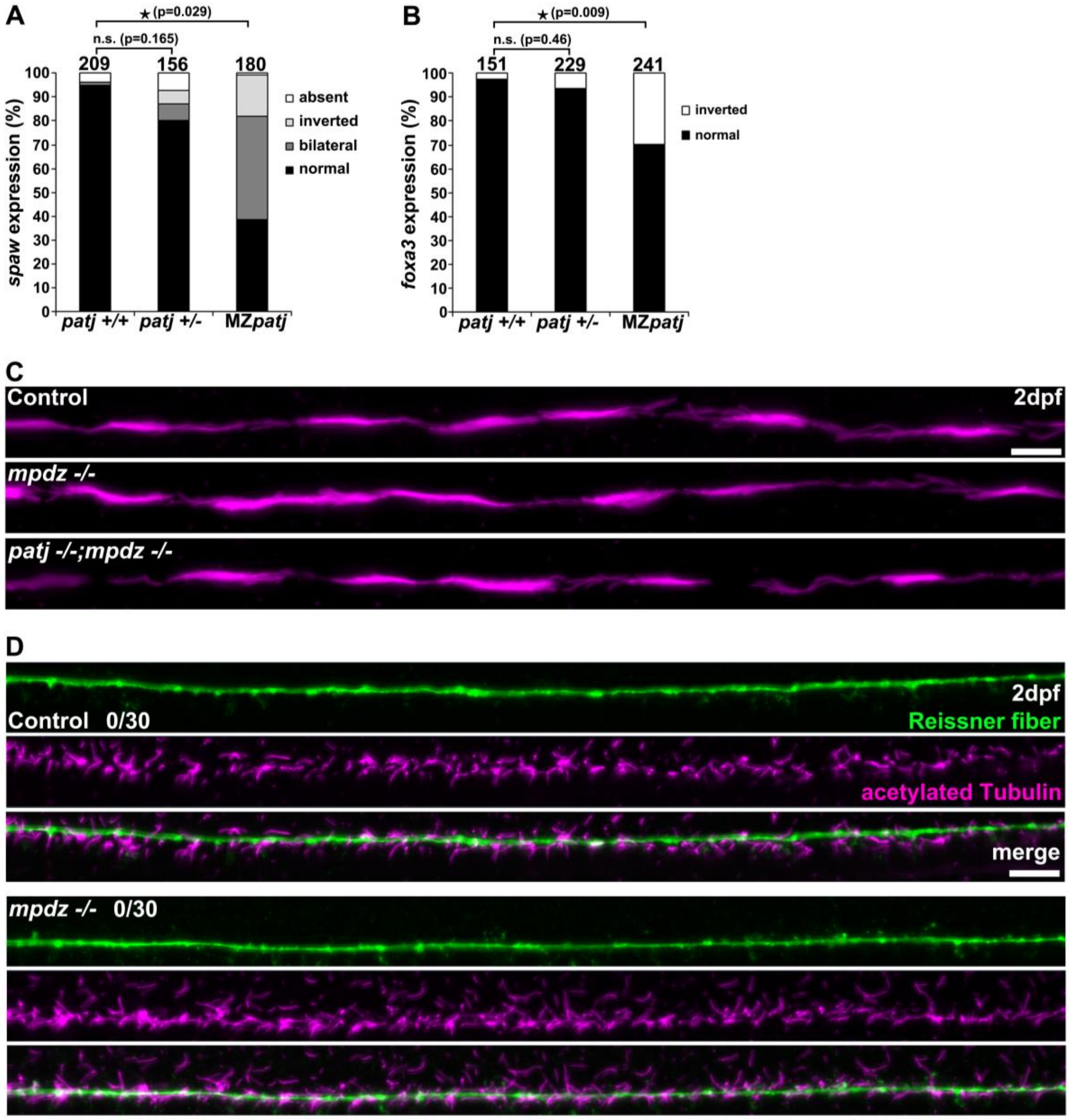

Figure S4. Analyses of cilia formation and ciliopathy-associated phenotypes in CRISPR/Cas9-induced *patj*, *mpdz* and *patj;mpdz* zebrafish mutants

(A) Quantification of WISH-analyzed *patj* +/+, *patj* +/- and MZ*patj* embryos at 18S using *southpaw* (*spaw*) as LR asymmetry marker. *Spaw* expression was analyzed in respect to its

localization in the embryo as normal, bilateral, inverted and absent. Number of embryos used for analyses are shown above respective bar. (B) Quantification of WISH-analyzed *patj* +/+, *patj* +/- and MZ*patj* embryos at 2dpf using *foxa3* as LR asymmetry marker. *Foxa3* expression was analyzed in respect to its localization in the embryo as normal, bilateral and inverted. Number of embryos used for analyses are shown above respective bar. (C) Representative confocal images of the pronephric tubule of a homozygous *mpdz* knockout, a double homozygous *patj;mpdz* knockout and respective control sibling embryo at 2dpf immunostained with anti-acetylated Tubulin as a ciliary marker. Scale bar: 10µm. (D) Representative confocal images of a homozygous *mpdz* knockout and respective control sibling embryo at 2dpf immunostained with anti-Reissner fiber (RF) and anti-acetylated Tubulin as a ciliary marker. Numbers represent embryos displaying RF disorganization and embryos that have been analyzed in total. Scale bar: 10µm.

Supplemental Materials and Methods

PCR-based methods

RNA was isolated from zebrafish embryos following the RNeasy manual (Qiagen), and cDNA synthesis was performed using the ProtoScript First Strand cDNA Synthesis kit (NEB). A 435bp PCR-product amplified with primers patj-F: 5`-CAGATCCCCAGTCTTCCAAA-3` and patj-R: 5`-ATCTCTGTACTGCGCCTCGT-3`was cloned into pCRII-TOPO (Thermo Fisher Scientific). For temporal *patj* expression analysis in zebrafish we performed semi-quantitative RT-PCR using following primers: patj-F, patj-R, ef1α-F: 5`-ATCTACAAATGCGGTGGAAT-3`and ef1α-R: 5`-ATACCAGCCTCAAACTCACC-3`. For validation of the *patj*-MO1 and *patj* -MO2 efficiency, we used patj-F2: 5`-CGACCGGGTGTTGTTTTCAT-3`, patj-R2: 5`-TGATCATTCTCCAGCAGCCT-3` and patj-F3: 5`-TCATGGTGTCTTTGTCCGAC-3`, patj-R3: 5`-TCTTTCCACCGACAATCCCA-3`, respectively, and ef1α-F, ef1α-R. Quantitative real-time PCR (qPCR) was performed as previously described [1]. Total RNA was obtained from 30 control or double homozygous *patj;mpdz* knockout embryos at 2dpf. *ef1α* was used as normalization control. Technical triplicates of five biological samples were analyzed for gene expression.

Morpholino injection

Morpholino oligonucleotide (MO) injection was performed as described [1] . Following splicing-blocking MOs (Gene Tools) were used: *patj*–MO1 (5′-GCGGCCCTGTCAGAAACAAAACACA-3′) and *patj*-MO2 (5′-

CCCTGCAGATATGAGGGATGATCAC-3′). We used a standard negative control MO (Co-MO (5'-CCTCTTACCTCAGTTACAATTTATA-3')) provided by Gene Tools.

Generation of *patj*, *mpdz* and *patj*;*mpdz* zebrafish knockouts

To knockout *patj* or *mpdz* in zebrafish, we used a gRNA targeting the genomic sequence 5`-GGTCTGAGACCAGAGGGGGTTGG-3` in exon5 of *patj* and a gRNA targeting the genomic sequence 5`-CCAGCGGTGCCTGCAGGCAGTGG-3` in *mpdz*. Therefore, we cloned double stranded oligos (patj_gRNA_F: 5`-TAGGGGTCTGAGACCAGAGGGGGT-3` and patj_gRNA_R: 5`-AAACACCCCCTCTGGTCTCAGACC-3`; mpdz_gRNA_F: 5`-TAGGCCAGCGGTGCCTGCAGGCAG-3` and mpdz_gRNA_R: 5`-AAACCTGCCTGCAGGCACCGCTGG-3`) into BsmBI linearized pT7-gRNA (Addgene). One nanoliter containing *Cas9* mRNA (500ng/µl) and either *patj-gRNA* (250ng/µl) or *mpdz-gRNA* (250ng/µl) was injected into one cell-stage *li1Tg* embryos. For our experiments, we used a maternal-zygotic (MZ) *patj* knockout with a deletion of one nucleotide in exon5 resulting in a frame-shift and premature stop codon. *Mpdz* mutants (deletion of seven nucleotides resulting in a frame-shift and premature stop codon) were crossed to MZ*patj* mutants to obtain double heterozygous *patj*;*mpdz* mutants.

Whole mount in situ hybridization (WISH) analysis and immunostaining

WISH and whole mount immunostaining procedures were performed as previously described [1]. The antibody directed against the Reissner fiber was a kind kift from Stéphane Gobron and used 1:200 in this study.

Microscopy and image analysis

Microscopy and image analysis was performed as previously described [1]. Measurement of ciliary length is described elsewhere [2].

Statistical analysis and quantification

Statistical analysis and quantification have been carried out as recently described [1].

Accession numbers

Zebrafish patj/Patj (NM_001127185.2/NP_001120657.2) and mpdz/Mpdz (XM_073907624.1/ XP_073763725.1).

Supplemental References

1. Ott, E., Hoff, S., Indorf, L., Ditengou, F.A., Muller, J., Renschler, G., Lienkamp, S.S., Kramer-Zucker, A., Bergmann, C., and Epting, D. (2023). A novel role for the chloride intracellular channel protein Clic5 in ciliary function. Sci Rep *13*, 17647. 10.1038/s41598-023-44235-y.
2. Epting, D., Senaratne, L.D.S., Ott, E., Holmgren, A., Sumathipala, D., Larsen, S.M., Wallmeier, J., Bracht, D., Frikstad, K.M., Crowley, S., et al. (2020). Loss of CBY1 results in a ciliopathy characterized by features of Joubert syndrome. Hum Mutat *41*, 2179-2194. 10.1002/humu.24127.
