# Supplementary material for: PATJ deficiency leads to cystic kidney disease and related ciliopathies
Source: HGG Adv. 2025 Sep 9;7(1):100514. doi: 10.1016/j.xhgg.2025.100514 (PMC12512994; doi:10.1016/j.xhgg.2025.100514)
Supplement: Document S2. Article plus supplemental information [file mmc2.pdf]

HGG Advances

BRIEF COMMUNICATION

PATJ deficiency leads to cystic kidney disease and related ciliopathies

Daniel Epting,[1,6,*] Daniela A. Braun,[2] Eva Decker,[3] Elisabeth Ott,[1] Tobias Eisenberger,[3] Nadine Bachmann,[3] Pavel Nedvetsky,[4] Michael P. Krahn,[4] Friedhelm Hildebrandt,[5] and Carsten Bergmann[1,3,*]

Summary

Cystic kidney disease and related ciliopathies are caused by pathogenic variants in genes that commonly result in ciliary dysfunction. For a substantial number of individuals affected by those cilia-related diseases, the causative gene remains unknown. Using massively parallel sequencing, we here identified a pathogenic bi-allelic variant in the gene encoding PALS1-associated tight junction protein ([PATJ] also known as inactivation-no-afterpotential D-like, INADL) in an individual with ciliopathy. The affected fetus carried the homozygous truncating *PATJ* nonsense variant c.830delC (p.Pro277fsX), and presented with a syndromic phenotype mainly characterized by polycystic kidney disease and hydrocephalus. Using zebrafish (*Danio rerio*) as a vertebrate *in vivo* model organism, we could validate our patient findings and demonstrated a ciliopathy phenotype. In addition, we were able to address a hitherto not described role of Patj for cilia formation and function. Taken together, with the Crumbs cell polarity complex member PATJ, we add a new member to the large family of ciliopathy-related human disease proteins that is different from the classical ciliopathy protein classes, and may offer new perspectives for drug development.

Introduction

Virtually all non-dividing vertebrate cells possess either non-motile primary cilia or motile cilia. These protrude from the outside of the cells, acting respectively as mechano-sensors or creating a fluid flow. Reports of the last two decades have clearly shown that cilia-related defects are responsible for a huge and still growing number of genotypically and phenotypically variable human disorders, collectively termed ciliopathies. Among these are autosomal dominant and recessive polycystic kidney disease (PKD) (ADPKD/ARPKD; MIM: PS173900), nephronopthisis (NPH) (MIM: PS256100), and a number of syndromic phenotypes such as Joubert syndrome (MIM: PS213300), Meckel syndrome (MIM: PS249000), and Bardet-Biedl syndrome (MIM: PS209900).[1] Many causative genes are pleiotropic, resulting in an extensive list of potential clinical features such as cystic kidney and liver disease, congenital hepatic fibrosis, obesity, intellectual disability, retinal degeneration, polydactyly, hydrocephalus, infertility, and situs inversus. Despite remarkable progress in the identification of disease-causing genes and their encoded proteins related to cystic kidney disease or related ciliopathies, the genetic cause for a significant number of ciliopathy-affected individuals is still unknown. Emerging evidence indicates that proteins traditionally associated with epithelial polarity also play an important role in the etiology of ciliopathies. Some members of the polarity complexes (Par and Crumbs complex) have, besides their well-described roles in apical-basal polarity and establishing/maintenance of cell-cell junctions, additional functions in cilia formation and function. Hence, Par complex members PAR3, PAR6, and aPKC co-localize at the primary cilium as well as the Crumbs complex member CRB3 (both isoforms CRB3A and CRB3B) and are required for proper cilia formation and function. Moreover, there is evidence that CRB3A interacts with the Par complex, thereby acting upstream of it and recruiting Par complex members to the cilium (reviewed in Bazellieres et al.[2]). Functional *in vivo* studies revealed that depletion of Crumbs proteins resulted in cilia abnormalities and ciliopathy-related phenotypes in zebrafish.[3,4] In addition, *Crb3* knockout mouse models demonstrated perinatal lethality and cilia-related phenotypes such as cystic kidneys and improper airway clearance.[5,6] The Crumbs complex member PATJ physically interacts and co-localizes with the nephrocystins NPHP1 and NPHP4 (both proteins are linked to NPH) that is probably important for epithelial morphogenesis.[7] In addition, a physical interaction of PATJ and polycystin-2 (PC2) (linked to ADPKD) was described to be crucial for the regulation of PC2 channel activity and thereby probably plays a role for ADPKD pathogenesis.[8] Only recently, it has been shown that PATJ depletion in kidney tubular epithelial cells results in disturbed apical-basal polarity, lumen formation in 3D cyst cultures, tight junction

[1]Department of Medicine IV, Faculty of Medicine, Medical Center-University of Freiburg, University of Freiburg, Freiburg, Germany; [2]Department Molecular Nephrology, Internal Medicine D (MedD), University Hospital of Münster (UKM), 48149 Münster, Germany; [3]Medizinische Genetik Mainz, Limbach Genetics, Mainz, Germany; [4]Medical Cell Biology, Medical Clinic D, University Hospital of Münster, Albert-Schweitzer Campus 1-A14, 48149 Münster, Germany; [5]Department of Pediatrics, Boston Children's Hospital, Harvard Medical School, Boston, MA 02115, USA
[6]Lead contact
*Correspondence: daniel.epting@uniklinik-freiburg.de (D.E.), carsten.bergmann@medgen-mainz.de (C.B.)
https://doi.org/10.1016/j.xhgg.2025.100514.
© 2025 The Author(s). Published by Elsevier Inc. on behalf of American Society of Human Genetics.
This is an open access article under the CC BY license (http://creativecommons.org/licenses/by/4.0/).

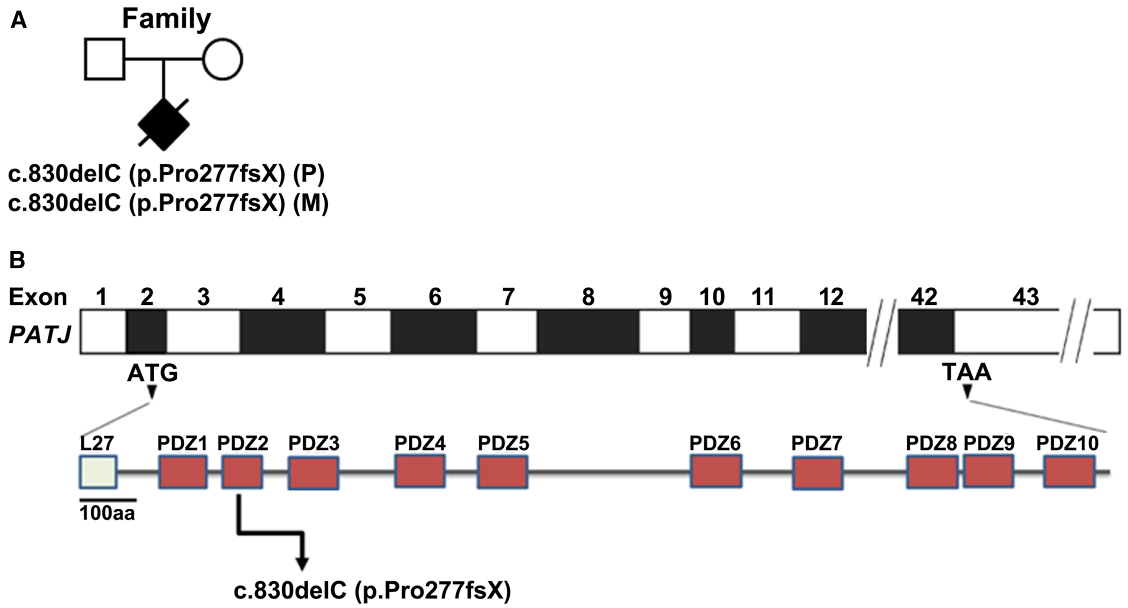

Figure 1. Identification of bi-allelic *PATJ* variants in an affected individual
(A) Pedigree for family with *PATJ* genetic variant c.830delC (p.Pro277fsX). Healthy parents and affected individual are shown in white and black boxes, respectively. *PATJ* genetic changes are shown below the symbol of the affected individual. The affected pregnancy was prenatally terminated (black symbol with transverse line); an autopsy was not performed.
(B) Schematic of human *PATJ* comprising 43 exons. Human *PATJ* encodes for a 1,801 amino acid protein with a single L27 domain and 10 PDZ domains (GenPept: NP_795352.3). The site of human *PATJ* variant identified is located in the second PDZ domain.

assembly, and cilia formation.[9] Mechanistically, this study demonstrated that PATJ binds to and inhibits HDAC7 thereby regulating primary cilia formation. Notably, this function of PATJ does not require the interaction with PALS1, suggesting a novel role of PATJ in cilia formation, which is distinct from its known function in the Crumbs complex. However, an *in vivo* role of PATJ in ciliogenesis is still lacking, and no *PATJ* (MIM: 603199) variants have been described in patients so far. Here, we report the identification of a homozygous truncating *PATJ* nonsense variant in an individual with cystic kidney disease and related ciliopathies. In addition, our analyses in zebrafish demonstrate ciliopathy-associated phenotypes upon Patj depletion and a role of Patj in cilia formation and function.

Material and methods

Genetic analysis

Research was performed following written informed consent and according to the declaration of Helsinki. DNA extraction was performed according to standard procedures. NGS technologies and comprehensive bioinformatic analyses utilized in this project are described in detail elsewhere.[10,11] Our approach is optimized in low-performance regions as well as in critical regions such as in *PKD1* as described.[12] High and reproducible coverage achieved by our sequencing approach also enabled copy-number variation (CNV) analysis. Performance of the wet-lab and bioinformatic processes are validated and controlled according to national and international guidelines reaching high sensitivity for SNV, Indels, and CNVs using well-established reference samples as well as a large cohort of positive controls, especially for CNVs.[13,14] For interpretation of identified variants, we have developed our own algorithms using a stepwise filtering process conducted by an experienced team of scientists and supported by various bioinformatics decision tools. Sequence variants of interest were verified by Sanger sequencing if NGS results failed internal validation guidelines.

Zebrafish husbandry, lines and embryo maintenance

The fish used in this study were maintained at the Zebrafish Facility of the Medical Center of the University of Freiburg. All animal work, zebrafish maintenance, and staging of embryos has been conducted as described recently.[15] The study was approved by the Institutional Animal Care of the Medical Center of the University of Freiburg and the Regional Council Freiburg (permit ID G-16/89). All methods were carried out in accordance with ARRIVE guidelines. The following wild-type (WT) and transgenic strains were used: AB/TL (WT), *li1Tg*,[16] cup^{tc321},[17] and $elipsa^{tp49d}$.[18]

Results

We performed massively parallel sequencing of the exome of an affected fetus with cystic kidney disease and hydrocephalus originating from a consanguineous marriage for which we could not identify any pathogenic variant in known genes prior to this. These analyses resulted in the identification of an allegedly pathogenic *PATJ* nonsense variant c.830delC (p.Pro277fsX) in the homozygous state (Figure 1).

In order to better understand the function of PATJ and to validate our patient findings, we used the model

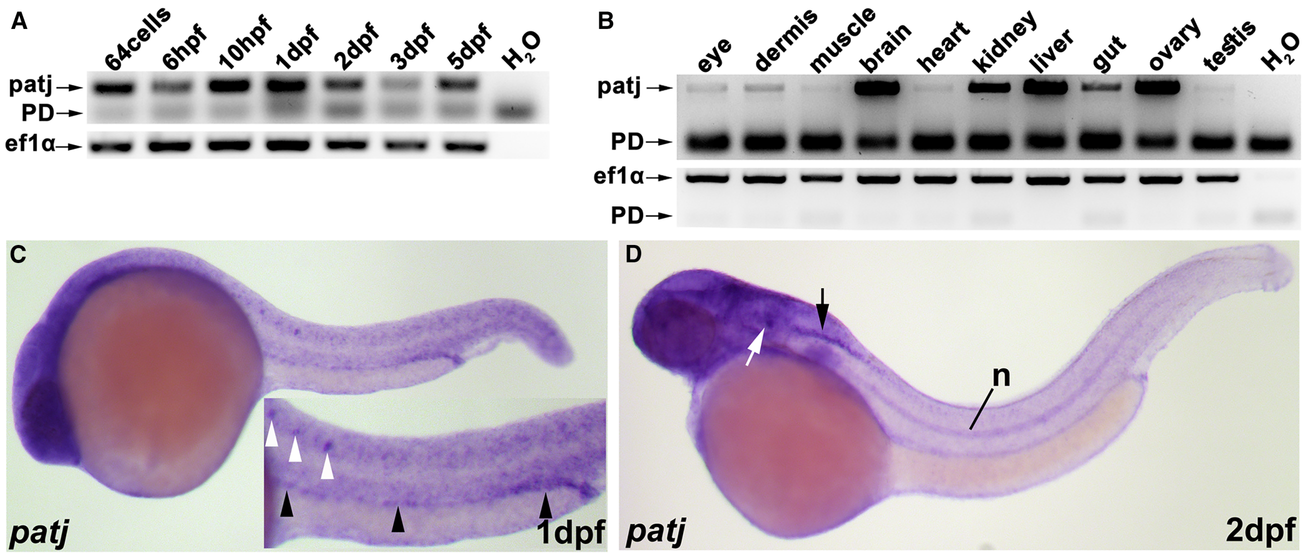

Figure 2. Expression analyses reveal specific expression of *patj* during embryogenesis and in adult organs in zebrafish
(A and B) Analyses of *patj* expression via semi-quantitative RT-PCR on cDNA of different embryonic developmental stages (A) or adult organs (B) in zebrafish, respectively. H_2O served as negative control and *ef1α* as loading control. PD, primer dimer.
(C and D) Analyses of *patj* expression via WISH at 1 and 2dpf; pronephric tubules (black arrowheads), neuronal cell populations in the spinal cord (white arrowheads), otic vesicle (white arrow), hindbrain (black arrow), and notochord (n). Embryos are shown from lateral with anterior to the left.

organism zebrafish, which offers unique advantages for analyzing various aspects of vertebrate development including ciliogenesis. We first analyzed the temporal and spatial expression of *patj* mRNA in zebrafish. To do this, we performed semi-quantitative reverse transcriptase-PCR (RT-PCR) on cDNA from different embryonic developmental stages and adult organs of zebrafish. These studies revealed that *patj* is expressed throughout early development and with different intensity levels in the analyzed organs with prominent expression in brain, kidney, liver, and ovary (Figures 2A and 2B). In addition, whole-mount *in situ* hybridization (WISH) analyses revealed expression of *patj* in highly ciliated tissues, e.g., pronephric tubules, spinal cord, and otic vesicles at 1 day post-fertilization (dpf) and 2 dpf (Figures 2C and 2D).

We also analyzed a potential ciliary role of Patj in zebrafish by using a morpholino (MO)-based knockdown approach. For this, we used two splicing-blocking MOs (*patj*-MO1 and *patj*-MO2), which, in zebrafish, respectively target intron4-exon5 and intron5-exon6 boundaries of *patj* pre-mRNA. The efficiency of MOs was validated by semi-quantitative RT-PCR, and showed, compared with the control, significantly reduced *patj* PCR-product levels in the Patj morphant embryos (Figure S1A). Knockdown with 4 ng of *patj*-MO1 or 6 ng of *patj*-MO2 resulted in significant hydrocephalus formation, otolith deposition defects, ventral body curvature, and a randomized heart looping (indicates defective left-right [LR] asymmetry) at 2 dpf, representing well-described ciliopathy-associated phenotypes in zebrafish (Figures 3A and S1B–S1G).[15] Additionally, we performed WISH analyses using the marker *southpaw* (*spaw*) as the earliest known LR asymmetry marker and *forkhead box protein a3* (*foxa3*) as a marker for primordial liver, pancreas, and intestine in zebrafish embryogenesis. These analyses clearly demonstrated defective LR asymmetry in Patj-deficient zebrafish embryos compared with control (Figures 3B and 3C). In zebrafish, the organ of laterality is known as the Kupffer's vesicle, containing cells with a single motile cilium. Several studies have shown that defective cilia in this organ ultimately lead to LR asymmetry defects.[19] Analyses of cilia formation in the Kupffer's vesicle at the stage of eight somites showed that the cilia in the Patj morphants were significantly reduced in length compared with control embryos (Figure 3D). Noteworthy, motile cilia formation in the pronephric tubules (containing single ciliated and multiciliated cells) of Patj morphants appeared unaffected compared with control embryos at 1 and 2 dpf (Figure S2).

To verify the results of our MO experiments, we generated a Patj knockout in zebrafish (deletion of 1 bp in exon5 of *patj* leading to a frameshift and a premature stop codon) using the CRISPR-Cas9 technology (Figure S3). Surprisingly, maternal-zygotic (MZ) *patj* mutants did not display all of the ciliopathy-associated phenotypes that we observed in the Patj morphants, i.e., hydrocephalus formation and ventral body curvature; however, we detected striking LR asymmetry defects, analyzed by heart looping and by WISH analyses with the markers *spaw* and *foxa3* (Figures 4A, S4A, and S4B). Subsequent analyses of cilia in the Kupffer's vesicle revealed, compared with controls, significant shorter cilia in the MZ*patj* mutant embryos (Figure 4A). Multi-PDZ

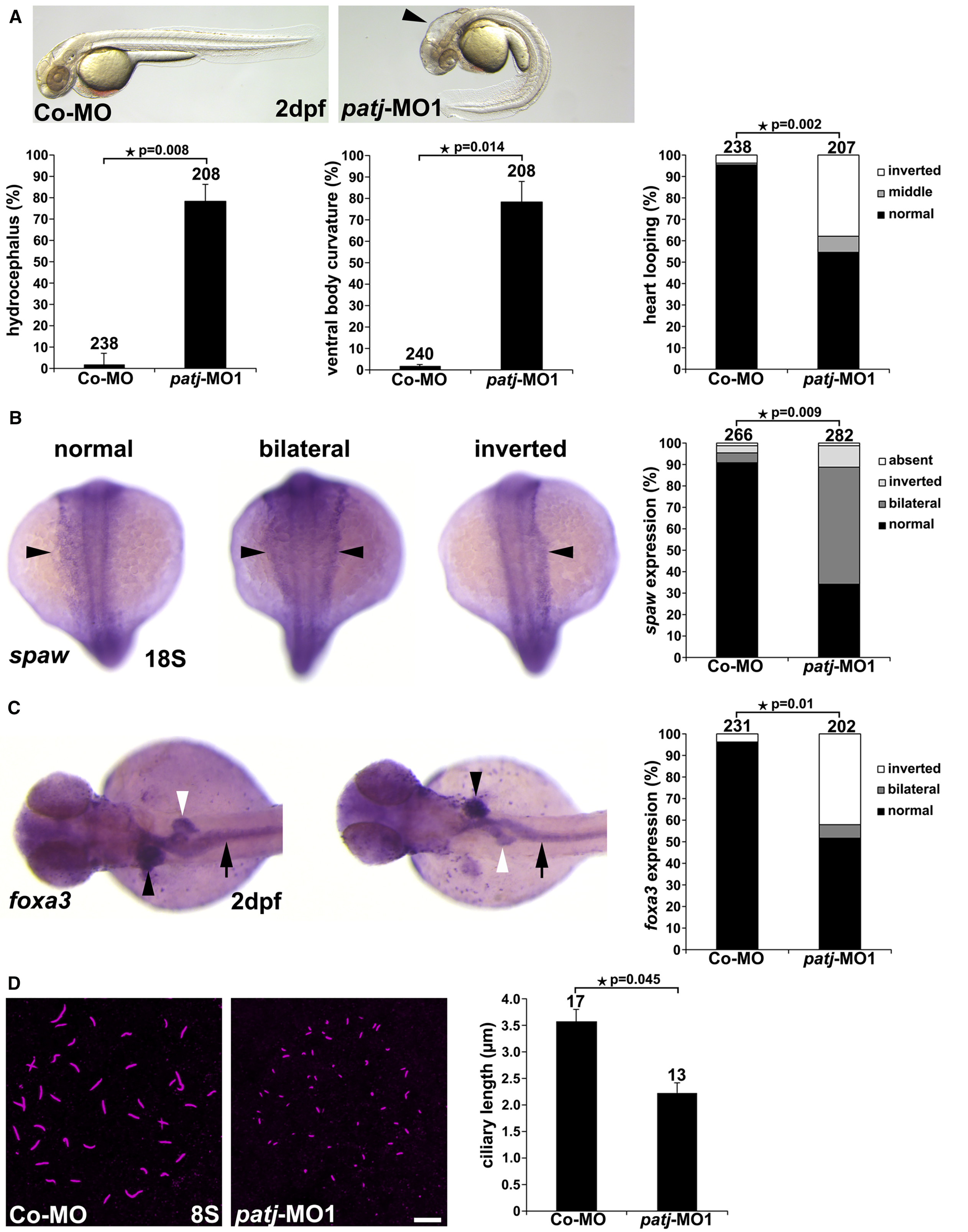

Figure 3. Knockdown of Patj results in ciliopathy-associated phenotypes in zebrafish
(A) Representative bright-field images of Co-MO- and *patj*-MO1-injected embryos at 2dpf; hydrocephalus (black arrowhead). Embryos are shown from lateral with anterior to the left. Quantification of hydrocephalus formation, ventral body curvature, and altered heart looping (analyzed as normal, middle (unlooped), and inverted) of Co-MO- and *patj*-MO1-injected embryos at 2dpf.

(legend continued on next page)

domain protein 1 (MUPP1) represents a homolog of PATJ and, due to their high similarity, both proteins might act in a redundant fashion. Since we only observed ciliopathy-associated defects in early development of MZ*patj* mutant embryos, we considered the possibility that Mpdz (the zebrafish ortholog of MUPP1) might compensate for the loss of Patj later in development. Therefore, we generated a knockout for *mpdz* in zebrafish via CRISPR-Cas9 (deletion of 7 bp in *mpdz* leading to a frameshift and a premature stop codon), and performed an incross of homozygous *mpdz* and MZ*patj* animals to generate double heterozygous *patj*;*mpdz* knockouts. Incrosses of animals with double heterozygous *patj*;*mpdz* knockout resulted in embryos with only a pericardial edema and others that show a dorsal body curvature in addition to the pericardial edema. Subsequent genotyping revealed, in accordance with Mendelian ratios, that the pericardial edema phenotype corresponds to homozygous deletion of Mpdz, whereas the phenotype with dorsal body curvature and pericardial edema results from the homozygous deletion of both proteins, Patj and Mpdz (Figure 4B). Cilia formation in the pronephric tubules of homozygous *mpdz* knockouts and double homozygous *patj*;*mpdz* knockouts appeared unaffected at 2 dpf (Figure S4C).

Recent studies in zebrafish revealed that proper body axis morphogenesis relies on cilia-dependent formation of the Reissner fiber (RF), an acellular and filamentous structure that is present in the cerebrospinal fluid.[20,21] We therefore analyzed RF formation in double homozygous *patj*;*mpdz* knockouts by whole-mount co-immunostaining using antibodies for acetylated tubulin and RF. As controls, we included the well-described cilia-defective mutants $elipsa^{tp49d}$ and cup^{tc321} that display ventral and dorsal body curvature, respectively.[17,18] Defective RF formation has been described for $elipsa^{tp49d}$, but respective results have not been reported for the cup^{tc321} so far.[20] Our results revealed RF disorganization in $elipsa^{tp49d}$, cup^{tc321}, and double homozygous *patj*;*mpdz* knockout embryos compared with homozygous *mpdz* knockout and control sibling embryos at 2 dpf (Figures 4C and S4D). Moreover, we performed quantitative RT-PCR to analyze cilia-dependent signaling pathways in double homozygous *patj*;*mpdz* knockouts. While the Wnt signaling pathway was unaffected, we observed, compared with the control, significant dysregulation of Hedgehog (Hh) signaling pathway components in double homozygous *patj*;*mpdz* knockout embryos compared with the control (Figure 4D).

Discussion

Our results conclusively identified *PATJ* as a novel cystic kidney disease and ciliopathy-related candidate gene. We report here a convincing bi-allelic nonsense *PATJ* variant in an individual affected by cystic kidney disease and related ciliopathies. The mutation results in a truncated version of the protein, most likely leading to a non-functional or degraded protein, which most probably explains the severity of the clinical manifestations. For the PATJ homolog MUPP1 (also known as MPDZ), pathogenic variants have been reported in affected individuals (from unrelated families) presenting with autosomal recessive non-syndromic congenial hydrocephalus.[22,23] In addition, mouse *Mpdz* knockout models lead to the formation of hydrocephalus.[24] Notably, this report revealed morphological intact cilia on ependymal cells but demonstrated that MUPP1/MPDZ is essential for maintaining ependymal integrity. Another report has shown that loss of MPDZ in mouse results in hyperpermeability of the choroid plexus causing hydrocephalus formation.[25] To date, no *Patj* mouse knockout model has been reported. We have studied in the *in vivo* model organism zebrafish a hitherto undescribed role of Patj in cilia formation and function. RT-PCR and WISH analyses showed that *patj* is highly expressed throughout embryogenesis and in different ciliated embryonic and adult tissues. MO-mediated knockdown of Patj resulted in well-known ciliopathy-associated phenotypes, and analyses of cilia in the Kupffer's vesicle (LR organizer in zebrafish) revealed a significant reduction of ciliary length and thus most probably causes the observed LR asymmetry defects. A CRISPR-Cas9-mediated knockout of Patj recapitulates the observed Patj morphant phenotypes, showing reduced ciliary length in the Kupffer's vesicle and LR asymmetry defects, but no other ciliopathy-associated phenotypes could be documented.

(B) Quantification of WISH-analyzed Co-MO- and *patj*-MO1-injected embryos at 18 somites (S) using *southpaw* (*spaw*) as LR asymmetry marker. *Spaw* expression was analyzed in respect to its localization in the embryo as normal (on the left side, black arrowhead), bilateral (on both sides, black arrowheads), inverted (on the right side, black arrowhead), and absent (no expression). Embryos are shown from dorsal with anterior to the top.
(C) Quantification of WISH-analyzed Co-MO- and *patj*-MO1-injected embryos at 2dpf using *foxa3* as LR asymmetry marker. *Foxa3* expression was analyzed in respect to its localization in the embryo as normal (liver [black arrowhead] on the left side, pancreas [white arrowhead] on the right side and normal looping of the intestine [black arrow]), bilateral (liver, pancreas, and intestine in the middle of embryonic axis), and inverted (liver [black arrowhead] on the right side, pancreas [white arrowhead] on the left side, and reversed looping of the intestine [black arrow]). Embryos are shown from dorsal with anterior to the left.
(D) Representative confocal images of the Kupffer's vesicle of Co-MO- and *patj*-MO1-injected embryos at the stage of 8S immunostained with anti-acetylated tubulin as a ciliary marker. Scale bar, 10 μm. Quantification of the ciliary length in the Kupffer's vesicle of Co-MO- and *patj*-MO1-injected embryos at 8S.
Number of embryos used for analyses are shown above respective bar. Data were analyzed by Student's *t* test (2-sided, unpaired); error bars represent the standard error of the mean (SEM). A *p* value of <0.05 was considered statistically significant.

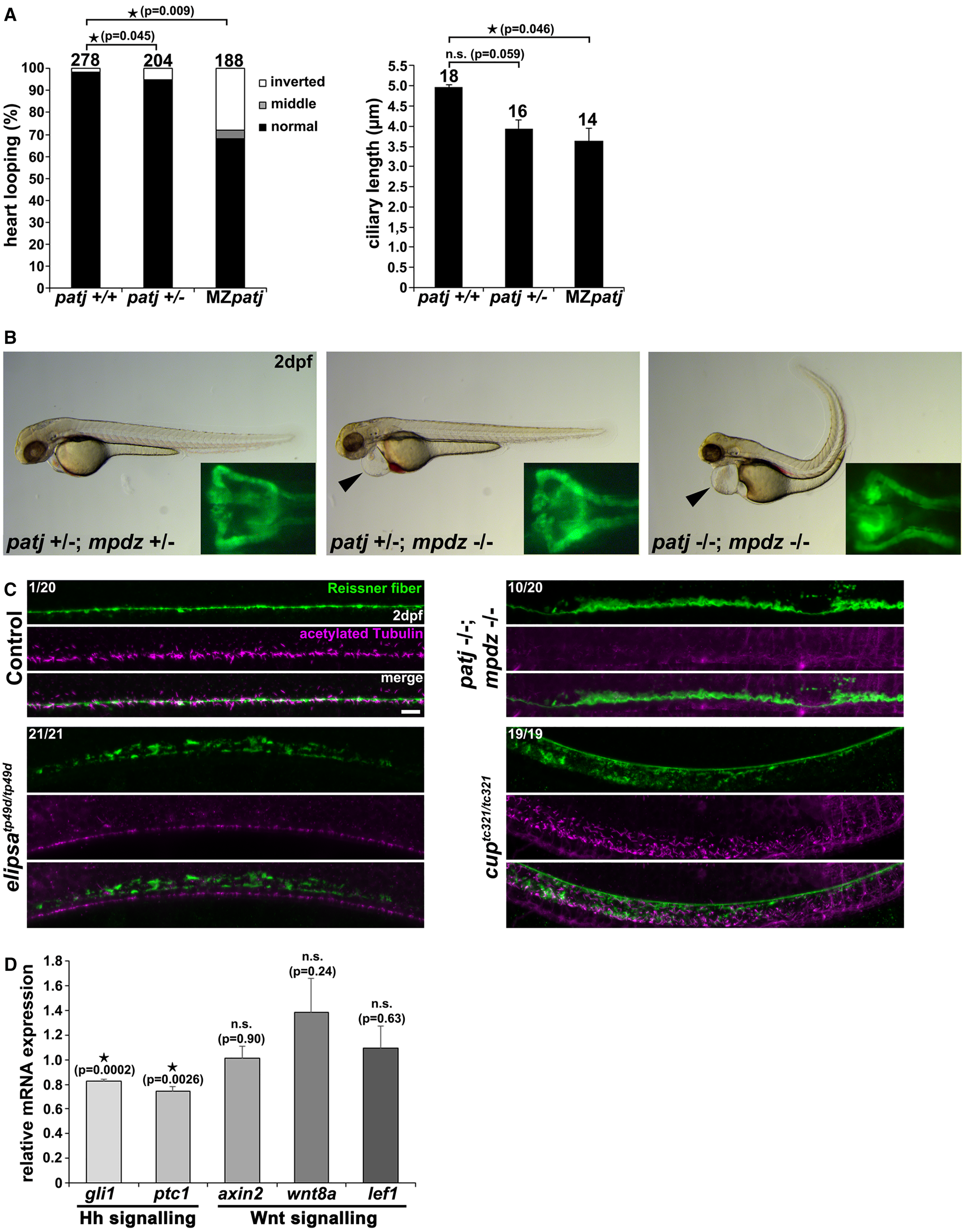

Figure 4. CRISPR-Cas9-induced *patj* and *patj;mpdz* zebrafish mutants display ciliopathy-associated phenotypes

(A) Quantification of altered heart looping (analyzed as normal, middle (unlooped) and inverted), and of the ciliary length in the Kupffer's vesicle of wild-type (*patj* +/+), heterozygous *patj* mutants (*patj* +/−), and MZ*patj* mutants at 2dpf and 8S, respectively. N-umber of embryos used for analyses are shown above respective bars. Data were analyzed by Student's *t*test (2-sided, unpaired); error bars represent the SEM. A *p* value of <0.05 was considered statistically significant.

(legend continued on next page)

Mechanisms of genetic compensatory response have been well studied in zebrafish, and thus it is reasonable that the Patj homolog Mpdz compensates for the loss of Patj in later development of *Patj* knockout embryos.[26,27] Notably, it has been reported that loss of MPDZ leads to increased expression of PATJ protein, and *in vitro* silencing of MPDZ resulted in increased *PATJ* mRNA expression.[24,28] A double homozygous *patj*;*mpdz* knockout displayed pericardial edema (due to homozygous deletion of Mpdz) and a dorsal body curvature in zebrafish. Hence, we observed phenotypic discrepancy regarding body curvature between Patj morphants and Patj;Mpdz mutants, presenting with ventral and dorsal body curvature, respectively. Possibly the Patj;Mpdz double knockout results in a yet unknown genetic compensation mechanism that might have an influence in body axis formation and probably results in this context in a dorsal curvature phenotype. Of note, compensation mechanisms and their investigation are likely to be complex and far from being completely understood.[29] Moreover, in Patj morphants and Patj;Mpdz mutants maternal *patj*/*mpdz* mRNA and/or Patj/Mpdz protein might also have an influence in the phenotypic variability that we observed in our knockdown and knockout approaches. The majority of reported ciliopathy-associated zebrafish morphants and mutants display a ventral body curvature, but also a dorsal body curvature was reported, e.g., Pc2 and Bicc1 loss-of-function.[17,30] Our analyses revealed defects in the formation of the RF in Pc2 and Patj;Mpdz double knockouts which are most probably a result of defective cilia formation and function in the floor plate. Future studies are needed to analyze whether Patj potentially interacts directly or indirectly with PKD disease proteins Pc2 and/or Bicc1. Defective primary cilia function often results in the dysregulation of important signaling pathway components. Indeed, we identified dysregulated expression of cilia-associated Hh signaling pathway components, thus further supporting an essential ciliary role of Patj. Our results provide further insight into the poorly characterized function of polarity complex members in cystic kidney disease and related ciliopathies, and might be useful for respective drug discovery and therapeutic approaches.

Data and code availability

Exome sequence data were generated during clinical testing; however, study individuals were not consented for data sharing. Other datasets used and/or analyzed during the current study are available from the corresponding authors on reasonable request.

Acknowledgments

We are grateful to the staff of the Aquatic Core Facility (AquaCore [RI_00544]) at the University Freiburg Medical Center – IMITATE, Germany, for the zebrafish care. We would like to thank the Life Imaging Center of the University Freiburg for the use of confocal microscopes and technical support. We thank Eric Barnsley for critical reading of the manuscript. M.P.K. receives support from the Deutsche Forschungsgemeinschaft (DFG, German Research Foundation) (KR3901/9-1, KR3901/9-2, SFB1348-A05, and TRR422). E.O. receives support from the DFG (Project-ID 431984000 – Collaborative Research Center SFB 1453). C.B. holds a part-time faculty appointment at the University of Freiburg in addition to his position as medical and managing partner and director of the Medizinische Genetik Mainz and Limbach Genetics. E.D., T.E., and N.B. are employees of the Medizinische Genetik Mainz. C.B. receives support from the DFG (BE 3910/8-1, BE 3910/8-2, BE 3910/9-1, and Project-ID 431984000 – Collaborative Research Center SFB 1453), the Federal Ministry of Education and Research (BMBF, 01GM1903I and 01GM1903G), and the European Union (EU HORIZON-HLTH-2022-DISEASE-06).

Declaration of interests

The authors declare no competing interests.

Supplemental information

Supplemental information can be found online at https://doi.org/10.1016/j.xhgg.2025.100514.

(B) Representative bright-field images of a homozygous *mpdz* mutant (*patj* +/−; *mpdz* −/−) displaying pericardial edema (black arrowhead) and of a double homozygous *patj*;*mpdz* mutant (*patj* −/−; *mpdz* −/−) displaying pericardial edema (black arrowhead) and dorsal body curvature compared with a respective control clutch embryo (*patj* +/−; *mpdz* +/−) at 2dpf. Quantitative analysis from randomly selected control clutch embryos (without phenotype) revealed following genotypes: 3x (*patj* +/+; *mpdz* +/+), 3x (*patj* +/+; *mpdz* +/−), 5x (*patj* +/−; *mpdz* +/+), 5x (*patj* +/−; *mpdz* +/−), 2x (*patj* −/−; *mpdz* +/+) and 6x (*patj* −/−; *mpdz* +/−). Quantitative analysis from randomly selected embryos displaying pericardial edema revealed following genotypes: 9x (*patj* +/+; *mpdz* −/−) and 15x (*patj* +/−; *mpdz* −/−). Quantitative analysis from randomly selected embryos displaying pericardial edema and dorsal body curvature revealed following genotypes: 24x (*patj* −/−; *mpdz* −/−). The respective inserts show a fluorescent image of EGFP expression of the same embryo (dorsal view) indicating no detectable pronephric cyst formation for all three different genotypes. Embryos are shown from lateral with anterior to the left.

(C) Representative confocal images of $cup^{tc321/tc321}$, $elipsa^{tp49d/tp49d}$, *patj* −/−; *mpdz* −/− mutant embryos and respective control sibling embryos at 2dpf immunostained with anti-RF and anti-acetylated tubulin as a ciliary marker. Numbers represent embryos displaying RF disorganization and embryos that have been analyzed in total. Scale bar, 10 μm.

(D) Quantitative RT-PCR analyses reveal unaltered expression of Wnt signaling components *axin2*, *wnt8a*, and *lef1* while Hh signaling components *gli1* and *ptc1* were significantly downregulated in double homozygous *patj;mpdz* mutant embryos compared with control sibling embryos at 2dpf ($E^{-\Delta\Delta CT}$, normalized to control samples for all genes). Data were analyzed with Graphpad Prism software and one sample *t* test; error bars represent the SEM.

Web resources

OMIM, http://www.omim.org/

Received: April 22, 2025
Accepted: September 5, 2025

References

1. Reiter, J.F., and Leroux, M.R. (2017). Genes and molecular pathways underpinning ciliopathies. Nat. Rev. Mol. Cell Biol. *18*, 533–547. https://doi.org/10.1038/nrm.2017.60.
2. Bazellieres, E., Aksenova, V., Barthelemy-Requin, M., Massey-Harroche, D., and Le Bivic, A. (2018). Role of the Crumbs proteins in ciliogenesis, cell migration and actin organization. Semin. Cell Dev. Biol. *81*, 13–20. https://doi.org/10.1016/j.semcdb.2017.10.018.
3. Omori, Y., and Malicki, J. (2006). oko meduzy and related crumbs genes are determinants of apical cell features in the vertebrate embryo. Curr. Biol. *16*, 945–957. https://doi.org/10.1016/j.cub.2006.03.058.
4. Hazime, K., and Malicki, J.J. (2017). Apico-basal Polarity Determinants Encoded by crumbs Genes Affect Ciliary Shaft Protein Composition, IFT Movement Dynamics, and Cilia Length. Genetics *207*, 1041–1051. https://doi.org/10.1534/genetics.117.300260.
5. Whiteman, E.L., Fan, S., Harder, J.L., Walton, K.D., Liu, C.J., Soofi, A., Fogg, V.C., Hershenson, M.B., Dressler, G.R., Deutsch, G.H., et al. (2014). Crumbs3 is essential for proper epithelial development and viability. Mol. Cell Biol. *34*, 43–56. https://doi.org/10.1128/MCB.00999-13.
6. Charrier, L.E., Loie, E., and Laprise, P. (2015). Mouse Crumbs3 sustains epithelial tissue morphogenesis in vivo. Sci. Rep. *5*, 17699. https://doi.org/10.1038/srep17699.
7. Delous, M., Hellman, N.E., Gaudé, H.M., Silbermann, F., Le Bivic, A., Salomon, R., Antignac, C., and Saunier, S. (2009). Nephrocystin-1 and nephrocystin-4 are required for epithelial morphogenesis and associate with PALS1/PATJ and Par6. Hum. Mol. Genet. *18*, 4711–4723. https://doi.org/10.1093/hmg/ddp434.
8. Duning, K., Rosenbusch, D., Schlüter, M.A., Tian, Y., Kunzelmann, K., Meyer, N., Schulze, U., Markoff, A., Pavenstädt, H., and Weide, T. (2010). Polycystin-2 activity is controlled by transcriptional coactivator with PDZ binding motif and PALS1-associated tight junction protein. J. Biol. Chem. *285*, 33584–33588. https://doi.org/10.1074/jbc.C110.146381.
9. Fiedler, J., Moennig, T., Hinrichs, J.H., Weber, A., Wagner, T., Hemmer, T., Schröter, R., Weide, T., Epting, D., Bergmann, C., et al. (2023). PATJ inhibits histone deacetylase 7 to control tight junction formation and cell polarity. Cell. Mol. Life Sci. *80*, 333. https://doi.org/10.1007/s00018-023-04994-3.
10. Lu, H., Galeano, M.C.R., Ott, E., Kaeslin, G., Kausalya, P.J., Kramer, C., Ortiz-Brüchle, N., Hilger, N., Metzis, V., Hiersche, M., et al. (2017). Mutations in DZIP1L, which encodes a ciliary-transition-zone protein, cause autosomal recessive polycystic kidney disease. Nat. Genet. *49*, 1025–1034. https://doi.org/10.1038/ng.3871.
11. Schrezenmeier, E., Kremerskothen, E., Halleck, F., Staeck, O., Liefeldt, L., Choi, M., Schüler, M., Weber, U., Bachmann, N., Grohmann, M., et al. (2021). The underestimated burden of monogenic kidney disease in adults waitlisted for kidney transplantation. Genet. Med. *23*, 1219–1224. https://doi.org/10.1038/s41436-021-01127-8.
12. Eisenberger, T., Decker, C., Hiersche, M., Hamann, R.C., Decker, E., Neuber, S., Frank, V., Bolz, H.J., Fehrenbach, H., Pape, L., et al. (2015). An efficient and comprehensive strategy for genetic diagnostics of polycystic kidney disease. PLoS One *10*, e0116680. https://doi.org/10.1371/journal.pone.0116680.
13. Rehm, H.L., Bale, S.J., Bayrak-Toydemir, P., Berg, J.S., Brown, K.K., Deignan, J.L., Friez, M.J., Funke, B.H., Hegde, M.R., Lyon, E.; and Working Group of the American College of Medical Genetics and Genomics Laboratory Quality Assurance Commitee (2013). ACMG clinical laboratory standards for next-generation sequencing. Genet. Med. *15*, 733–747. https://doi.org/10.1038/gim.2013.92.
14. Matthijs, G., Souche, E., Alders, M., Corveleyn, A., Eck, S., Feenstra, I., Race, V., Sistermans, E., Sturm, M., Weiss, M., et al. (2016). Guidelines for diagnostic next-generation sequencing. Eur. J. Hum. Genet. *24*, 1515. https://doi.org/10.1038/ejhg.2016.63.
15. Ott, E., Hoff, S., Indorf, L., Ditengou, F.A., Müller, J., Renschler, G., Lienkamp, S.S., Kramer-Zucker, A., Bergmann, C., and Epting, D. (2023). A novel role for the chloride intracellular channel protein Clic5 in ciliary function. Sci. Rep. *13*, 17647. https://doi.org/10.1038/s41598-023-44235-y.
16. Perner, B., Englert, C., and Bollig, F. (2007). The Wilms tumor genes wt1a and wt1b control different steps during formation of the zebrafish pronephros. Dev. Biol. *309*, 87–96. https://doi.org/10.1016/j.ydbio.2007.06.022.
17. Schottenfeld, J., Sullivan-Brown, J., and Burdine, R.D. (2007). Zebrafish curly up encodes a Pkd2 ortholog that restricts left-side-specific expression of southpaw. Development *134*, 1605–1615. https://doi.org/10.1242/dev.02827.
18. Omori, Y., Zhao, C., Saras, A., Mukhopadhyay, S., Kim, W., Furukawa, T., Sengupta, P., Veraksa, A., and Malicki, J. (2008). Elipsa is an early determinant of ciliogenesis that links the IFT particle to membrane-associated small GTPase Rab8. Nat. Cell Biol. *10*, 437–444. https://doi.org/10.1038/ncb1706.
19. Forrest, K., Barricella, A.C., Pohar, S.A., Hinman, A.M., and Amack, J.D. (2022). Understanding laterality disorders and the left-right organizer: Insights from zebrafish. Front. Cell Dev. Biol. *10*, 1035513. https://doi.org/10.3389/fcell.2022.1035513.
20. Cantaut-Belarif, Y., Sternberg, J.R., Thouvenin, O., Wyart, C., and Bardet, P.L. (2018). The Reissner Fiber in the Cerebrospinal Fluid Controls Morphogenesis of the Body Axis. Curr. Biol. *28*, 2479–2486.e4. https://doi.org/10.1016/j.cub.2018.05.079.
21. Troutwine, B.R., Gontarz, P., Konjikusic, M.J., Minowa, R., Monstad-Rios, A., Sepich, D.S., Kwon, R.Y., Solnica-Krezel, L., and Gray, R.S. (2020). The Reissner Fiber Is Highly Dynamic In Vivo and Controls Morphogenesis of the Spine. Curr. Biol. *30*, 2353–2362.e3. https://doi.org/10.1016/j.cub.2020.04.015.
22. Al-Dosari, M.S., Al-Owain, M., Tulbah, M., Kurdi, W., Adly, N., Al-Hemidan, A., Masoodi, T.A., Albash, B., and Alkuraya, F.S. (2013). Mutation in MPDZ causes severe congenital hydrocephalus. J. Med. Genet. *50*, 54–58. https://doi.org/10.1136/jmedgenet-2012-101294.
23. Shaheen, R., Sebai, M.A., Patel, N., Ewida, N., Kurdi, W., Altweijri, I., Sogaty, S., Almardawi, E., Seidahmed, M.Z., Alnemri, A., et al. (2017). The genetic landscape of familial

congenital hydrocephalus. Ann. Neurol. *81*, 890–897. https://doi.org/10.1002/ana.24964.
24. Feldner, A., Adam, M.G., Tetzlaff, F., Moll, I., Komljenovic, D., Sahm, F., Bäuerle, T., Ishikawa, H., Schroten, H., Korff, T., et al. (2017). Loss of Mpdz impairs ependymal cell integrity leading to perinatal-onset hydrocephalus in mice. EMBO Mol. Med. *9*, 890–905. https://doi.org/10.15252/emmm.201606430.
25. Yang, J., Simonneau, C., Kilker, R., Oakley, L., Byrne, M.D., Nichtova, Z., Stefanescu, I., Pardeep-Kumar, F., Tripathi, S., Londin, E., et al. (2019). Murine MPDZ-linked hydrocephalus is caused by hyperpermeability of the choroid plexus. EMBO Mol. Med. *11*, e9540. https://doi.org/10.15252/emmm.201809540.
26. Rouf, M.A., Wen, L., Mahendra, Y., Wang, J., Zhang, K., Liang, S., Wang, Y., Li, Z., Wang, Y., and Wang, G. (2023). The recent advances and future perspectives of genetic compensation studies in the zebrafish model. Genes Dis. *10*, 468–479. https://doi.org/10.1016/j.gendis.2021.12.003.
27. Adachi, M., Hamazaki, Y., Kobayashi, Y., Itoh, M., Tsukita, S., Furuse, M., and Tsukita, S. (2009). Similar and distinct properties of MUPP1 and Patj, two homologous PDZ domain-containing tight-junction proteins. Mol. Cell Biol. *29*, 2372–2389. https://doi.org/10.1128/MCB.01505-08.
28. Assemat, E., Crost, E., Ponserre, M., Wijnholds, J., Le Bivic, A., and Massey-Harroche, D. (2013). The multi-PDZ domain protein-1 (MUPP-1) expression regulates cellular levels of the PALS-1/PATJ polarity complex. Exp. Cell Res. *319*, 2514–2525. https://doi.org/10.1016/j.yexcr.2013.07.011.
29. Peng, J. (2019). Gene redundancy and gene compensation: An updated view. J Genet Genomics *46*, 329–333. https://doi.org/10.1016/j.jgg.2019.07.001.
30. Bouvrette, D.J., Sittaramane, V., Heidel, J.R., Chandrasekhar, A., and Bryda, E.C. (2010). Knockdown of bicaudal C in zebrafish (Danio rerio) causes cystic kidneys: a nonmammalian model of polycystic kidney disease. Comp. Med. *60*, 96–106.

HGGA, Volume 7

Supplemental information

PATJ deficiency leads to cystic

kidney disease and related ciliopathies

Daniel Epting, Daniela A. Braun, Eva Decker, Elisabeth Ott, Tobias Eisenberger, Nadine Bachmann, Pavel Nedvetsky, Michael P. Krahn, Friedhelm Hildebrandt, and Carsten Bergmann

Supplemental information

Table of content:

Figure S1. Analyses of Patj knockdown efficiency and knockdown with *patj*-MO2 results in ciliopathy-associated phenotypes in zebrafish

Figure S2. Analyses of ciliogenesis in the pronephric tubules of Patj knockdown embryos reveals unaffected cilia formation

Figure S3. Generation of CRISPR/Cas9-induced *patj, mpdz* and *patj;mpdz* zebrafish mutants

Figure S4. Analyses of cilia formation and ciliopathy-associated phenotypes in CRISPR/Cas9-induced *patj, mpdz* and *patj;mpdz* zebrafish mutants

Supplemental Materials and Methods

Supplemental References

Figure S1

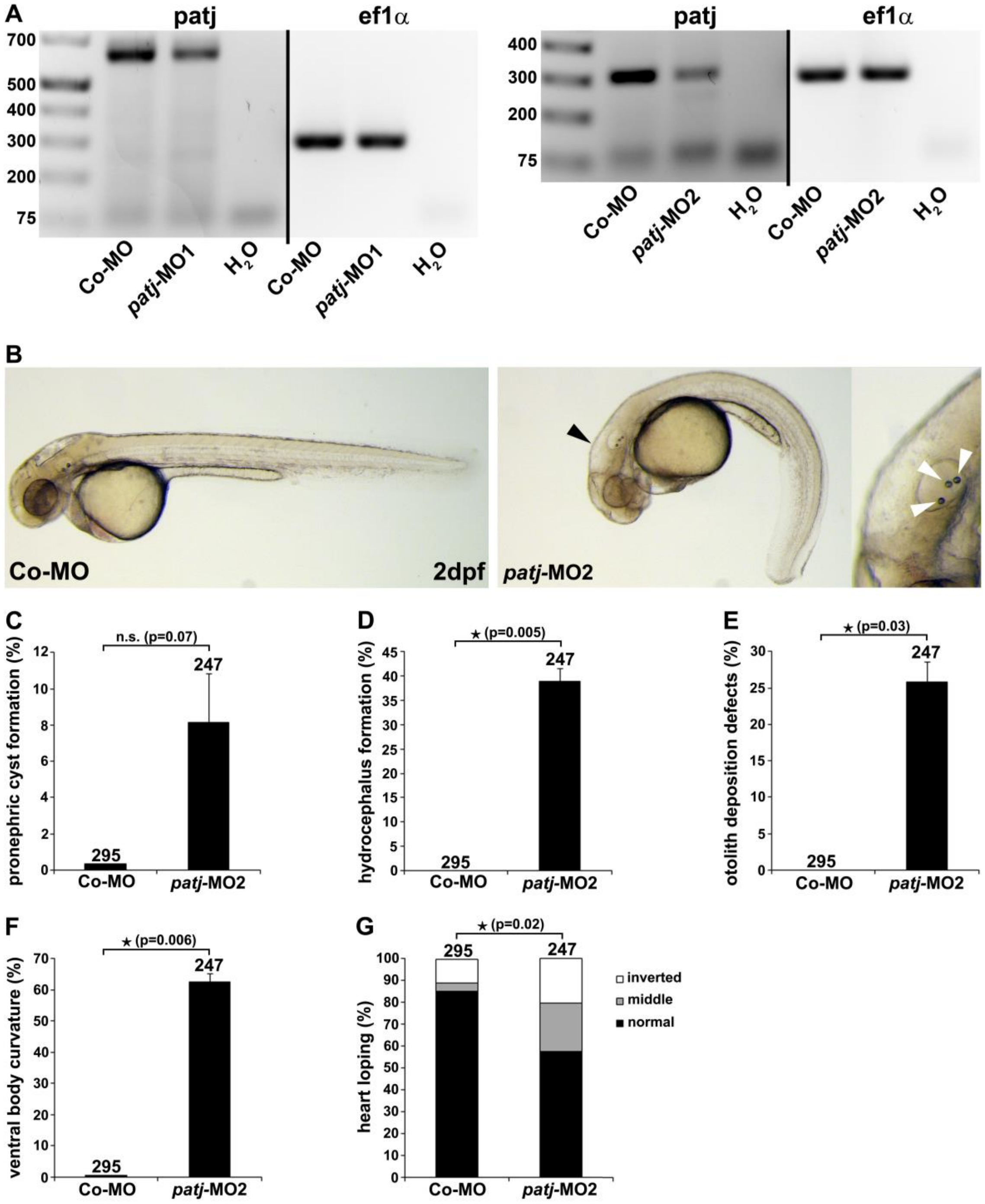

Figure S1. Analyses of Patj knockdown efficiency and knockdown with *patj*-MO2 results in ciliopathy-associated phenotypes in zebrafish

(A) RT-PCR reveals knockdown efficiency of splice-blocking MOs *patj*-MO1 and *patj*-MO2. Injection of either *patj*-MO1 or *patj*-MO2 results in significant reduced *patj* PCR-product level in the respective Patj morphant embryos compared to the control. H_2O served as negative control and *ef1α* as loading control. (B) Representative bright-field images of Co-MO and *patj*-MO2 injected embryos at 2dpf. In comparison to Co-MO injected embryos, injection of *patj*-

MO2 leads to hydrocephalus formation (black arrowhead), ventral body curvature and otolith deposition defects (white arrowheads indicate otoliths shown in a higher magnification of the otic vesicle; a typical defect is the absence of otoliths or the presence of one or three otoliths instead of two). Embryos are shown from lateral with anterior to the left. (C-G) Quantification of pronephric cyst formation (C), hydrocephalus formation (D), otolith deposition defects (E), ventral body curvature (F) and altered heart looping (analyzed as normal, middle (unlooped), inverted) (G) of embryos injected with Co-MO or *patj*-MO2 at 2dpf; number of embryos used for analyses are shown above each respective bar.

Figure S2

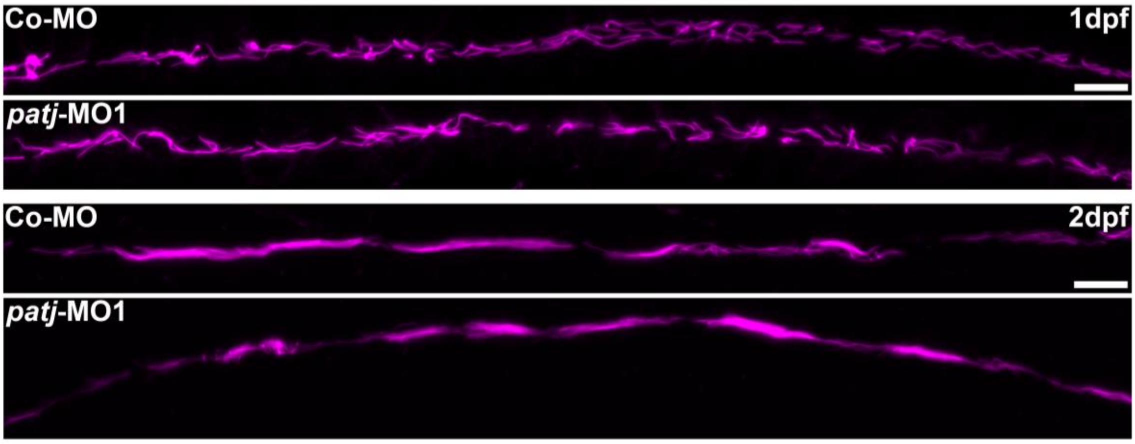

Figure S2. Analyses of ciliogenesis in the pronephric tubules of Patj knockdown embryos reveals unaffected cilia formation

Representative confocal images of the pronephric tubule of Co-MO and *patj*-MO1 injected embryos at 1 and 2dpf immunostained with anti-acetylated Tubulin as a ciliary marker. Scale bar: 10µm.

Figure S3

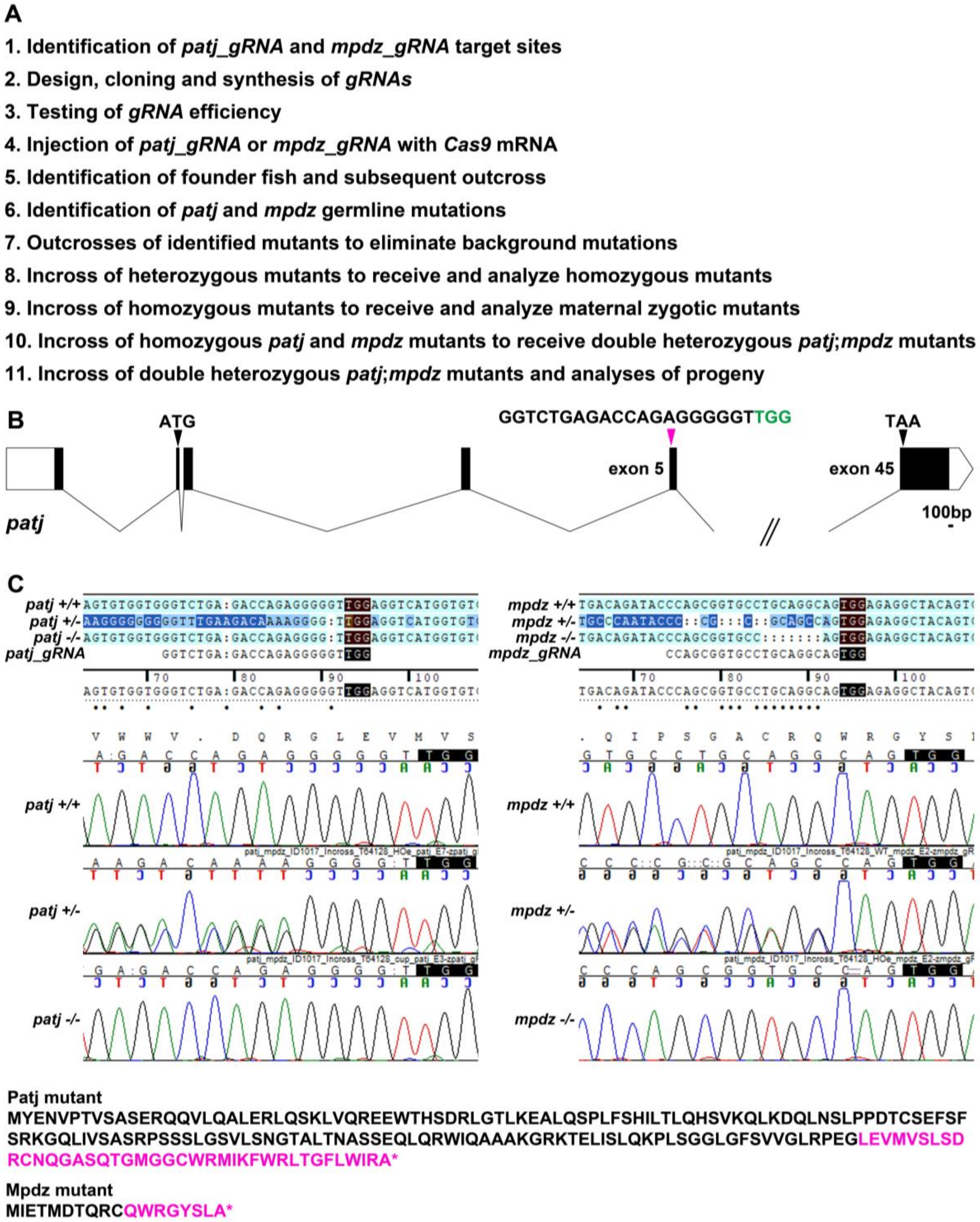

Figure S3. Generation of CRISPR/Cas9-induced *patj*, *mpdz* and *patj;mpdz* zebrafish mutants

(A) Flowchart summarizing the generation of CRISPR/Cas9-induced *patj* and *mpdz* single mutants and *patj;mpdz* double mutants in zebrafish. (B) Exon-intron structure of zebrafish *patj* (ENSDART00000132829.3) with the start codon ATG and stop codon TAA (black arrowheads) and the *patj_gRNA* target site (magenta arrowhead). (C) Sanger sequencing confirmed the deletion of nucleotide G in heterozygous and homozygous *patj* zebrafish mutants

compared to the respective control. Sanger sequencing confirmed the deletion of nucleotides TGCAGGC in heterozygous and homozygous *mpdz* zebrafish mutants compared to the respective control. The deletion of the nucleotide G in exon 5 of zebrafish *patj* results in a frameshift and premature stop codon in Patj mutants. The deletion of the nucleotides TGCAGGC in zebrafish *mpdz* (ENSDART00000160523.2) results in a frameshift and premature stop codon in Mpdz mutants.

Figure S4

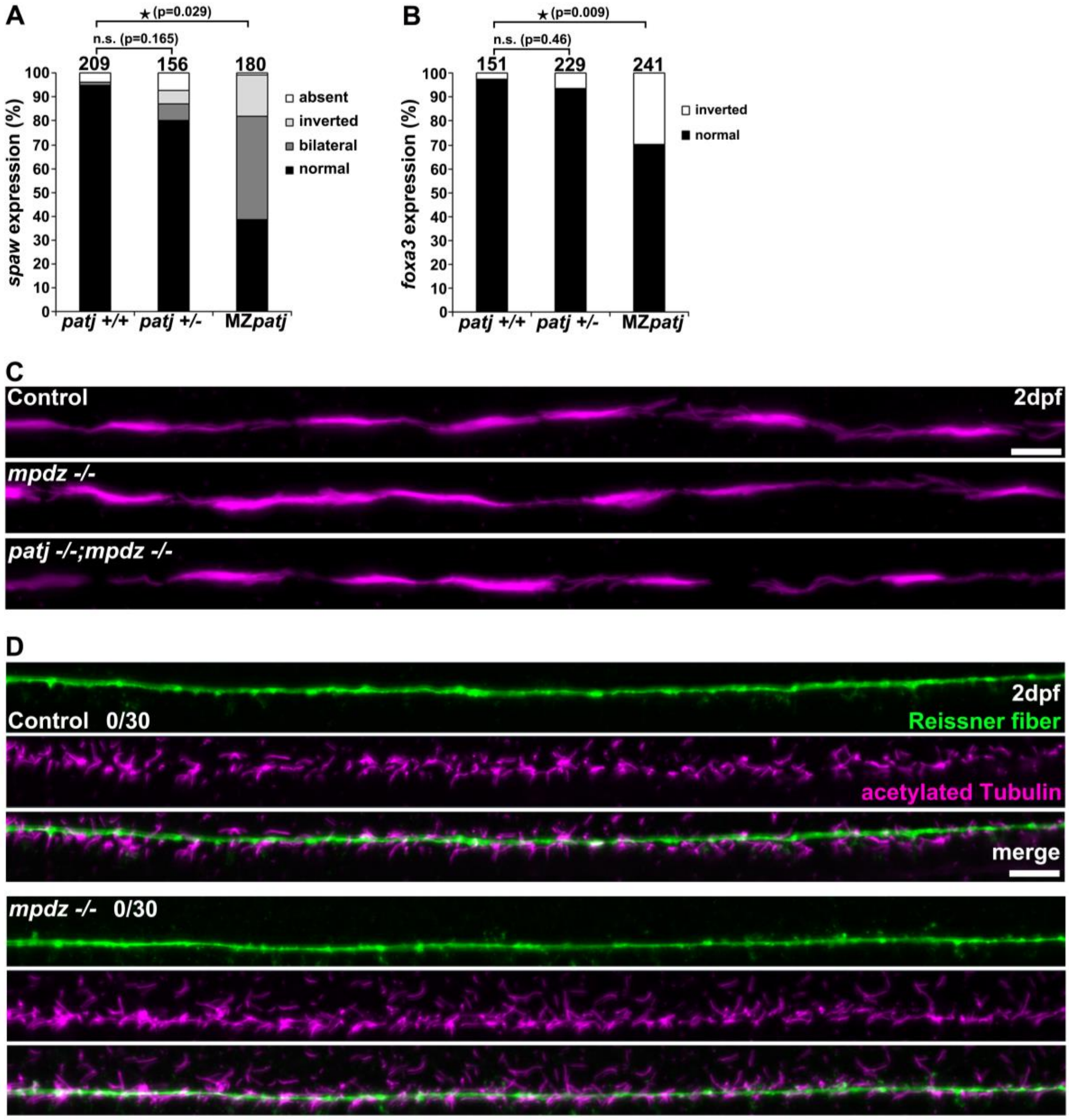

Figure S4. Analyses of cilia formation and ciliopathy-associated phenotypes in CRISPR/Cas9-induced *patj*, *mpdz* and *patj;mpdz* zebrafish mutants

(A) Quantification of WISH-analyzed *patj* +/+, *patj* +/- and MZ*patj* embryos at 18S using *southpaw* (*spaw*) as LR asymmetry marker. *Spaw* expression was analyzed in respect to its

localization in the embryo as normal, bilateral, inverted and absent. Number of embryos used for analyses are shown above respective bar. (B) Quantification of WISH-analyzed *patj* +/+, *patj* +/- and MZ*patj* embryos at 2dpf using *foxa3* as LR asymmetry marker. *Foxa3* expression was analyzed in respect to its localization in the embryo as normal, bilateral and inverted. Number of embryos used for analyses are shown above respective bar. (C) Representative confocal images of the pronephric tubule of a homozygous *mpdz* knockout, a double homozygous *patj;mpdz* knockout and respective control sibling embryo at 2dpf immunostained with anti-acetylated Tubulin as a ciliary marker. Scale bar: 10µm. (D) Representative confocal images of a homozygous *mpdz* knockout and respective control sibling embryo at 2dpf immunostained with anti-Reissner fiber (RF) and anti-acetylated Tubulin as a ciliary marker. Numbers represent embryos displaying RF disorganization and embryos that have been analyzed in total. Scale bar: 10µm.

Supplemental Materials and Methods

PCR-based methods

RNA was isolated from zebrafish embryos following the RNeasy manual (Qiagen), and cDNA synthesis was performed using the ProtoScript First Strand cDNA Synthesis kit (NEB). A 435bp PCR-product amplified with primers patj-F: 5`-CAGATCCCCAGTCTTCCAAA-3` and patj-R: 5`-ATCTCTGTACTGCGCCTCGT-3`was cloned into pCRII-TOPO (Thermo Fisher Scientific). For temporal *patj* expression analysis in zebrafish we performed semi-quantitative RT-PCR using following primers: patj-F, patj-R, ef1α-F: 5`-ATCTACAAATGCGGTGGAAT-3`and ef1α-R: 5`-ATACCAGCCTCAAACTCACC-3`. For validation of the *patj*-MO1 and *patj* -MO2 efficiency, we used patj-F2: 5`-CGACCGGGTGTTGTTTTCAT-3`, patj-R2: 5`-TGATCATTCTCCAGCAGCCT-3` and patj-F3: 5`-TCATGGTGTCTTTGTCCGAC-3`, patj-R3: 5`-TCTTTCCACCGACAATCCCA-3`, respectively, and ef1α-F, ef1α-R. Quantitative real-time PCR (qPCR) was performed as previously described [1]. Total RNA was obtained from 30 control or double homozygous *patj;mpdz* knockout embryos at 2dpf. *ef1α* was used as normalization control. Technical triplicates of five biological samples were analyzed for gene expression.

Morpholino injection

Morpholino oligonucleotide (MO) injection was performed as described [1]. Following splicing-blocking MOs (Gene Tools) were used: *patj*–MO1 (5′-GCGGCCCTGTCAGAAACAAAACACA-3′) and *patj*-MO2 (5′-

CCCTGCAGATATGAGGGATGATCAC-3′). We used a standard negative control MO (Co-MO (5'-CCTCTTACCTCAGTTACAATTTATA-3')) provided by Gene Tools.

Generation of *patj*, *mpdz* and *patj*;*mpdz* zebrafish knockouts

To knockout *patj* or *mpdz* in zebrafish, we used a gRNA targeting the genomic sequence 5`-GGTCTGAGACCAGAGGGGGTTGG-3` in exon5 of *patj* and a gRNA targeting the genomic sequence 5`-CCAGCGGTGCCTGCAGGCAGTGG-3` in *mpdz*. Therefore, we cloned double stranded oligos (patj_gRNA_F: 5`-TAGGGGTCTGAGACCAGAGGGGGT-3` and patj_gRNA_R: 5`-AAACACCCCCTCTGGTCTCAGACC-3`; mpdz_gRNA_F: 5`-TAGGCCAGCGGTGCCTGCAGGCAG-3` and mpdz_gRNA_R: 5`-AAACCTGCCTGCAGGCACCGCTGG-3`) into BsmBI linearized pT7-gRNA (Addgene). One nanoliter containing *Cas9* mRNA (500ng/µl) and either *patj-gRNA* (250ng/µl) or *mpdz-gRNA* (250ng/µl) was injected into one cell-stage *li1Tg* embryos. For our experiments, we used a maternal-zygotic (MZ) *patj* knockout with a deletion of one nucleotide in exon5 resulting in a frame-shift and premature stop codon. *Mpdz* mutants (deletion of seven nucleotides resulting in a frame-shift and premature stop codon) were crossed to MZ*patj* mutants to obtain double heterozygous *patj*;*mpdz* mutants.

Whole mount in situ hybridization (WISH) analysis and immunostaining

WISH and whole mount immunostaining procedures were performed as previously described [1]. The antibody directed against the Reissner fiber was a kind kift from Stéphane Gobron and used 1:200 in this study.

Microscopy and image analysis

Microscopy and image analysis was performed as previously described [1]. Measurement of ciliary length is described elsewhere [2].

Statistical analysis and quantification

Statistical analysis and quantification have been carried out as recently described [1].

Accession numbers

Zebrafish patj/Patj (NM_001127185.2/NP_001120657.2) and mpdz/Mpdz (XM_073907624.1/ XP_073763725.1).

Supplemental References

1. Ott, E., Hoff, S., Indorf, L., Ditengou, F.A., Muller, J., Renschler, G., Lienkamp, S.S., Kramer-Zucker, A., Bergmann, C., and Epting, D. (2023). A novel role for the chloride intracellular channel protein Clic5 in ciliary function. Sci Rep *13*, 17647. 10.1038/s41598-023-44235-y.
2. Epting, D., Senaratne, L.D.S., Ott, E., Holmgren, A., Sumathipala, D., Larsen, S.M., Wallmeier, J., Bracht, D., Frikstad, K.M., Crowley, S., et al. (2020). Loss of CBY1 results in a ciliopathy characterized by features of Joubert syndrome. Hum Mutat *41*, 2179-2194. 10.1002/humu.24127.
